# CCDC25: precise navigator for neutrophil extracellular traps on the prometastatic road

**DOI:** 10.1038/s41392-020-00285-6

**Published:** 2020-08-24

**Authors:** Ruochen Liu, Erhu Zhao, Feng Wang, Hongjuan Cui

**Affiliations:** 1 State Key Laboratory of Silkworm Genome Biology, College of Biotechnology, Southwest University, Chongqing, China; 2 Cancer Center, Medical Research Institute, Southwest University, Chongqing, China

**Keywords:** Metastasis, Cancer

**Fig. 1:**
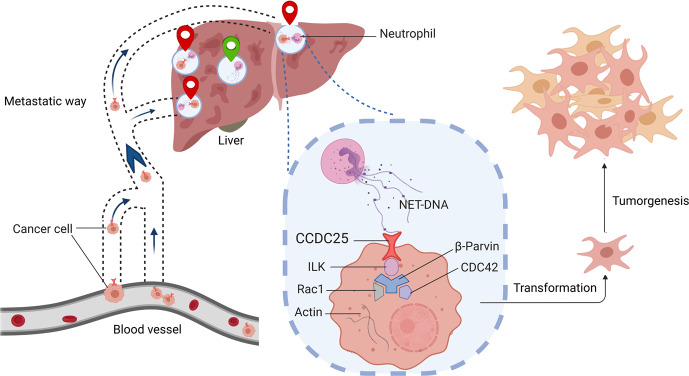
NET-DNA recognized by the plasma membrane protein CCDC25 promotes cancer metastasis. Neutrophil at distant tissue (liver) extrude NETs. The DNA sensor CCDC25 on the surface of cancer cells binds to the NET-DNA and subsequently activating the ILK–β-parvin–RAC1–CDC42 cascade, which induce cytoskeleton remodeling, migration, adhesion and proliferation of the cancer cells

The dissemination of cancer cells called metastasis accounts for the majority of deaths of cancer patients. Neutrophil extracellular traps (NETs) released by neutrophils to fight pathogens have been shown to promote metastasis in mouse models. However, the mechanism behind how NETs boost metastasis remains elusive. In a recent study in Nature,^1^ Yang et al reported that NETs are abundant in the liver metastases of patients with breast and colon cancers, and that the risk of liver metastasis in patients with early-stage breast cancer can be predicted by monitoring the levels of serum NETs. Furthermore, Yang et al.^1^ elucidated that the DNA component of NETs (NET-DNA) acts as a chemotactic factor that is recognized by coiled-coil domain containing protein 25 (CCDC25), a cytoplasmic membrane-localized extracellular DNA sensor of cancer cells, which in turn activates the integrin-linked kinase (ILK)–β-parvin–RAC1–CDC42 cascade to promote metastasis (Fig. 1). The clinical importance of CCDC25 was confirmed by Yang et al.,^1^ and targeting CCDC25 inhibits NET-mediated metastasis in mouse models, suggesting a potential therapeutic strategy to halt the metastasis.

NETs are chromatin DNA filaments decorated with cytotoxic proteins and proteases, and their formation by neutrophils is called NETosis. To study the clinical involvement of NETs in cancer development, Yang et al.^1^ evaluated NET formation in 544 patients with breast cancer using specific markers for neutrophils and NETosis. The results showed that NETs were scarce in the primary tumors but abundant in the liver metastases. Importantly, the authors found that liver metastases, but not metastases in other organs, were associated with higher levels of NET-DNA in the serum of patients with breast and colon cancers, indicating that monitoring NET-DNA in blood samples could predict the occurrence of liver metastases.

Using mouse models transplanted with breast or colon cancer cells of human or mouse origin, Yang and colleagues^1^ showed that NETosis was induced in the livers of the model mice before metastases could be detected. Deletion of NETs, by either directly degrading NET-DNA using DNaseI or generating mice deficient in an enzyme required for NETosis (peptidylarginine deiminase 4, PAD 4), significantly reduced liver metastases from mouse breast cancer cells. These data indicate that NETs play a crucial role in the process of liver metastasis from breast and colon cancers.

The mechanism underlying the prometastatic effect of NETs is a focus of research.^2,3^ Cools-Lartigue et al.^4^ proposed that the prometastatic function of NETs relies on merely trapping circulating cancer cells. Albrengues et al.^5^ reported that NET-associated proteases contribute to cancer development in distant tissues through proteolytic remodeling of the extracellular matrix and subsequent activation of integrin-mediated signaling in cancer cells. Yang et al.^1^ demonstrated that NET-DNA functions as a chemotactic factor that directly stimulates the migration, adhesion and proliferation of cancer cells.

An important unanswered question regarding NET-mediated prometastasis is how NETs are sensed by cancer cells. The team next sought to identify potential NET-DNA receptors on the plasma membrane of cancer cells.^1^ Using biotinylated NET-DNA as bait, CCDC25 was identified. In vitro DNA binding assays illustrated that CCDC25 preferentially bound NET-DNA with a high level of 8-hydroxy-2’-deoxyguanosine (8-OHdG, a hallmark of NET-DNA). Furthermore, they found that AA_21-25_ domain at the extracellular N terminus of CCDC25 was essential for this recognition.

The important role of CCDC25 in the NET-mediated metastasis of cancer cells was demonstrated by Yang and colleagues^1^ through multiple experiments: (1) Proliferation, adhesion and migration towards NET-DNA in vitro were inhibited when *CCDC25* gene was deleted from human breast cancer cells, while overexpression of CCDC25 promoted these processes; (2) *CCDC25* knockout significantly decreased NET-mediated lung metastases induced by nasal instillation of lipopolysaccharide (LPS)^5^ and liver metastases, while overexpression of CCDC25 significantly increased lung and liver metastases; (3) Similar in vitro and in vivo results were observed for human colon cancer cells and primary breast cancer cells; (4) Lung metastases upon LPS treatment were markedly reduced in hybrid mice with spontaneously formed breast cancer which were generated by crossing *CCDC25*-knockout mice with MMTV-PyMT mice; (5) Polyclonal antibody against CCDC25 efficiently blocked the NET-induced migration and adhesion of cancer cells in vitro and prevented the formation of liver metastases of human breast cancer cells in mouse models; (6) Clinically, the expression of CCDC25 in primary breast and colon cancer cells was associated with poor prognosis in patients, and CCDC25 closely interacted with NETs in the liver metastases of patients with breast cancer.

Finally, Yang et al.^1^ illustrated how the interaction between CCDC25 and NET-DNA promotes metastasis. Using His-tagged CCDC25 as bait in a pull-down assay, they identified a CCDC25-interacting protein, integrin-linked kinase (ILK). Further immunoprecipitation assays and genetic experiments revealed that the ILK–β-parvin–RAC1–CDC42 cascade acts downstream of CCDC25 to mediate NET-DNA stimulated liver metastasis (Fig. 1).

Collectively, Yang and colleagues provide new insights into the molecular mechanisms underlying NET-mediated cancer spread. A plasma membrane-located extracellular DNA sensor, CCDC25, has now come into focus and represents an appealing therapeutic target. Future studies will be required to evaluate the anticancer feasibility of targeting this protein. The expression profile of CCDC25 in different cell types and its potential functions in normal cells should be investigated. Since the authors have identified the specific part of CCDC25 responsible for its interaction with NET-DNA, there is an opportunity to develop specific inhibitors to interfere with this interaction without affecting the anti-infection functions of NETs. Moreover, although why NETs preferentially form in the liver over other metastatic sites remains to be elucidated, these findings by Yang and colleagues indicate that monitoring NET-DNA in blood samples could be used to predict liver metastasis.
